# Severe Vitamin B12 Deficiency in Pregnancy Mimicking HELLP Syndrome

**DOI:** 10.1155/2019/4325647

**Published:** 2019-03-25

**Authors:** Shravya Govindappagari, Michelle Nguyen, Megha Gupta, Ramy M. Hanna, Richard M. Burwick

**Affiliations:** ^1^Department of Obstetrics and Gynecology, Division of Maternal-Fetal Medicine, Cedars-Sinai Medical Center, Los Angeles, CA, USA; ^2^Department of Obstetrics and Gynecology, Adventist Health White Memorial, Los Angeles, CA, USA; ^3^Department of Obstetrics and Gynecology, Division of Maternal-Fetal Medicine, University of Texas Health Science Center at Houston, Houston, TX, USA; ^4^Department of Medicine, Division of Nephrology, UCLA David Geffen School of Medicine, Los Angeles, CA, USA; ^5^Department of Obstetrics and Gynecology, UCLA David Geffen School of Medicine, Los Angeles, CA, USA

## Abstract

Severe vitamin B12 deficiency may present with hematologic abnormalities that mimic thrombotic microangiopathy disorders such as hemolysis, elevated liver enzymes, and low platelet count (HELLP) syndrome. We report a patient diagnosed with severe vitamin B12 deficiency, following termination of pregnancy for suspected preeclampsia and HELLP syndrome at 21 weeks' gestation. When hemolysis and thrombocytopenia persisted after delivery, testing was performed to rule out other etiologies of thrombotic microangiopathy, including atypical hemolytic uremic syndrome, thrombotic thrombocytopenic purpura, and vitamin B12 deficiency. This work-up revealed undetectable vitamin B12 levels and presence of intrinsic factor antibodies, consistent with pernicious anemia. Parenteral B12 supplementation was initiated, with subsequent improvement in hematologic parameters. Our case emphasizes the importance of screening for B12 deficiency in pregnancy, especially in at-risk women with unexplained anemia or thrombocytopenia. Moreover, providers should consider B12 deficiency and pernicious anemia in the differential diagnosis of pregnancy-associated thrombotic microangiopathy.

## 1. Introduction

Mild vitamin B12 deficiency is common in pregnancy and due to increased fetal demand over gestation, 38% of women have low B12 levels by the time of delivery [1]. Early recognition is critical because low B12 levels in pregnancy have been associated with neural tube defects, preterm birth, and low neonatal birthweight [2, 3]. Heightened suspicion is warranted in women with risk factors for B12 deficiency, including obesity, prior bariatric surgery, inflammatory bowel disease, Helicobacter pylori infection, use of metformin or proton pump inhibitors, and certain vegan diets. Severe B12 deficiency may also result from pernicious anemia, which is caused by intrinsic factor blocking antibodies that interfere with B12 absorption.

In pregnancy, B12 deficiency may go unrecognized if anemia is mistakenly attributed to other causes such as iron deficiency or physiologic hemodilution. When severe B12 deficiency goes untreated, it can have profound effects, including severe anemia, peripheral neuropathy, cognitive decline, and a variety of neuropsychiatric manifestations. It may also manifest with microangiopathic hemolytic anemia and thrombocytopenia, mimicking other thrombotic microangiopathy (TMA) disorders such as atypical hemolytic uremic syndrome (aHUS), hemolysis, elevated liver enzymes, low platelet count (HELLP) syndrome, and thrombotic thrombocytopenic purpura (TTP) [4–6]. When TMA occurs in pregnancy, it is critical that B12 deficiency be ruled out because the treatment approach (B12 supplementation) varies greatly from other TMA disorders.

Here, we report the case of a patient diagnosed with pernicious anemia, following pregnancy termination for suspected preeclampsia and HELLP syndrome at 21 weeks' gestation.

## 2. Case Presentation

A 40-year-old multiparous African American woman at 21 weeks' 4-day gestation, with known chronic hypertension, was transferred to our medical center for management of hypertensive emergency. Prior to transfer, the patient had marked blood pressure elevation (peak 192/129 mmHg) and laboratory evaluation notable for hemoglobin 11.0 g/dl, platelet count 66 k/*μ*l, alanine transaminase (ALT) 20 U/L, aspartate transaminase (AST) 40 U/L, and creatinine 0.7 mg/dl (Table 1 for laboratory trends and reference range). Urine dipstick detected 4+ protein, 4+ blood, and +nitrites, and urine drug screen was positive for methamphetamines and marijuana. Intravenous ceftriaxone was given empirically for urinary tract infection.

On arrival to our medical center, blood pressure peaked at 205/114 mmHg and laboratory findings were like those noted above. In addition, lactate dehydrogenase (LDH) level was 985 U/L, with haptoglobin <8 mg/dl and 3 schistocytes per high power field on peripheral smear. Urine protein/creatinine ratio was 2.61 mg/mg (normal <0.3 mg/mg). Estimated fetal weight by ultrasound was 451g, which was appropriate for her gestational age. We suspect that methamphetamine use precipitated her hypertensive crisis, but the clinical picture and laboratory findings were concerning for severe preeclampsia and HELLP syndrome. Due to her previable gestational age and the life-threatening nature of her condition, termination of pregnancy was recommended, and she agreed. Magnesium sulfate was initiated for seizure prophylaxis, and she was managed in the intensive care unit on an intravenous nicardipine drip. Within 12 hours of her first misoprostol dose for labor induction, she had a precipitous vaginal delivery of a nonviable female neonate. Pathology examination of the placenta revealed decidual arteriopathy and accelerated villous maturation, which are findings reflective of severe hypertension and placental hypoxia.

Following delivery, severe hypertension (≥160/110 mmHg) persisted throughout the postpartum period despite triple agent antihypertensive therapy. On postpartum day 5, she continued to have severe range blood pressures, but declined inpatient stay and was discharged home on an oral regimen of chlorthalidone (50mg daily), amlodipine (10mg daily), and telmisartan (80mg daily). During her postpartum course in the hospital, patient had a work-up for secondary causes of hypertension. She had maternal echo showing mild left ventricular hypertrophy, but normal left ventricular ejection fraction (69%) and no evidence of aortic coarctation. She had normal renal artery Dopplers, ruling out renal artery stenosis. Serum aldosterone/renin ratio was normal, ruling out primary aldosteronism. She did not complete a 24-hour urine collection to measure total metanephrines and therefore pheochromocytoma was not formally ruled out. We suspect that methamphetamine use precipitated her hypertensive emergency, but blood pressures remained severe despite 7 days of inpatient observation and drug cessation.

TMA also persisted despite termination of pregnancy and drug cessation. On postpartum day 1 laboratory values were as follows: hemoglobin 8.4 g/dl, platelet count 90 k/*μ*l, LDH 706 U/L, haptoglobin <8 mg/dl, 2-3 schistocytes per high power field on smear, and reticulocyte count 4.6% (normal 0.5-2.0%). Her reticulocyte production index was noted to be suboptimal at 2.1% (normal >3.0%), after adjustment for low hematocrit (30.5%) [1]. Considering that the patient's liver enzymes remained normal after delivery despite ongoing hemolysis and thrombocytopenia, the diagnosis of HELLP syndrome was questioned. Alternative TMA etiologies were considered, including aHUS, TTP, and vitamin B12 deficiency. Serum creatinine peaked at 0.9 mg/dl and aHUS genetic panel was negative for disease-associated complement gene variants (Machaon Diagnostics), making a diagnosis of aHUS less likely. ADAMTS13 activity was 75% (normal >67%), making a diagnosis of TTP less likely. Meanwhile, vitamin B12 level was undetectable <146 *ρ*g/ml (normal 213-816 *ρ*g/ml) and intrinsic factor blocking antibodies were positive, consistent with pernicious anemia. Mean corpuscular volume was paradoxically low at 75.3 FL (normal 80-100 FL), and other causes of anemia were ruled out with normal iron panel, hemoglobin electrophoresis, and folate level.

Following diagnosis of pernicious anemia, the patient was started on subcutaneous B12 injections at a dose of 1000 mcg daily. She received 5 injections of vitamin B12 during her postpartum hospital stay. While blood pressures remained severely elevated on the day of discharge (postpartum day 5), laboratory parameters showed signs of improvement: Hemoglobin 9.5 g/dl, platelet count 175 k/*μ*l, and LDH 404 U/L (Table 1 for laboratory trends). The patient was discharged home with a plan for continued B12 supplementation and close outpatient follow up. At 2 weeks postpartum the patient had normal blood pressure (127/82) and was clinically well, but she declined additional laboratory testing.

## 3. Discussion

In pregnancy, TMA is most often secondary to preeclampsia or HELLP syndrome [7]. However, our case emphasizes the need to consider other etiologies in the differential diagnosis of TMA. It is critical to rule out TTP, aHUS, and B12 deficiency, because these disorders have established, nondelivery approaches to treatment. TTP is best treated with plasma exchange, aHUS with complement blockade, and B12 deficiency with B12 supplementation. For preeclampsia and HELLP syndrome, delivery is the only definitive treatment. Therefore, if delivery does not lead to TMA resolution, other etiologies should be carefully evaluated. TTP is less likely with a normal ADAMTS13 activity level, while the diagnosis of aHUS is less likely without renal failure or complement gene mutation. Vitamin B12 deficiency is initially considered with a low B12 level, but should be confirmed by metabolite testing (homocysteine, methylmalonic acid levels) or antibody testing (intrinsic factor blocking antibody, parietal cell antibody). In our case, positive intrinsic factor antibodies confirmed the diagnosis of pernicious anemia.

Providers may not immediately recognize B12 deficiency as a life-threatening condition, but it should be noted that pernicious anemia was fatal before B12 treatments were available. Vitamin B12 plays a critical role in DNA synthesis, and hematopoietic precursor cells are highly sensitive to abnormal DNA synthesis caused by B12 deficiency. While mild anemia, leukopenia, and thrombocytopenia are common, approximately 10% of patients with B12 deficiency develop life-threatening hematologic manifestations [8]. When erythropoiesis becomes ineffective, there is premature death of precursor red blood cells in the bone marrow, prior to their release in circulation. Cytoskeletal fragility predisposes to erythrocyte fragmentation, and laboratory findings of hemolysis include elevated LDH and low haptoglobin [4, 5]. This has been termed intramedullary hemolysis or pseudo-TMA and is also associated with low reticulocyte count or low reticulocyte production index. In the adult population, it is estimated that TMA accounts for 2-3% of the hematologic disorders related to B12 deficiency [8]. In addition, pernicious anemia accounts for most of these TMA cases, whereas TMA is less common with B12 deficiency secondary to malabsorption [4, 5].

In real-world practice, cases are complex with overlapping features, and we recognize that our case was no exception. Our patient had a positive urine drug screen for methamphetamines, and this likely precipitated her hypertensive crisis. The patient was also pregnant, and her clinical picture was worrisome for preeclampsia and HELLP syndrome. In this scenario, the safest and most prudent option is expedited delivery. However, it is worth noting that our patient had undiagnosed and untreated B12 deficiency, which may have contributed to her clinical picture. She did not have peripheral neuropathy or neuropsychiatric manifestations of B12 deficiency, but hematologic abnormalities can occur in the absence of neurologic abnormalities [9]. Considering the association between pernicious anemia and TMA, it is possible that severe B12 deficiency led directly to her hemolysis and thrombocytopenia. While termination of pregnancy may have been unavoidable, we want to emphasize the importance of recognizing and treating pernicious anemia. In our case, we suspect that B12 supplementation not only helped to improve laboratory parameters in the short-term, but also improved her long-term prognosis.

Our case emphasizes the importance of screening for B12 deficiency in pregnancy, especially in at-risk women with unexplained anemia or thrombocytopenia. Moreover, providers should consider B12 deficiency and pernicious anemia in the differential diagnosis of pregnancy-associated TMA.

## Figures and Tables

**Table 1:** Laboratory or treatment measures before and after delivery.

Laboratory or Treatment Measure*∗*	Initial evaluation	Prior to delivery	PPD 1	PPD 2	PPD 3	PPD4	PPD5
	21+4/7 weeks gestation	21+5/7 weeks gestation					
Hemoglobin (g/dl)	11.0	9.7	8.4	9.0	9.5	8.8	9.3
Platelet Count (k/*μ*l)	66	93	90	103	133	112	175
Lactate dehydrogenase (U/L)	985	872	706	n/a	n/a	n/a	404
Alanine transaminase (U/L)	20	14	24	12	12	13	15
Aspartate transaminase (U/L)	40	30	20	19	15	19	19
Creatinine (mg/dl)	0.7	0.9	0.9	0.6	0.8	0.8	0.8
Haptoglobin (mg/dl)	<8	n/a	<8	<8	n/a	n/a	n/a
Vitamin B12 (*ρ*g/ml)	n/a	n/a	<146	n/a	n/a	n/a	n/a
Vitamin B12 supplementation (subcutaneous injection)	n/a	n/a	1000 mcg	1000 mcg	1000 mcg	1000 mcg	1000 mcg

PPD: postpartum day; n/a: not available.

*∗* Normal ranges: Hemoglobin (11.6-15.4 g/dl); Platelet count (150-450 k/*μ*l); Lactate dehydrogenase (125-220); Alanine transaminase (0-55 U/L); Aspartate transaminase (5-34 U/L); Creatinine (0.6-1.1 mg/dl); Haptoglobin (30-200 mg/dl); Vitamin B12 (213-816 *ρ*g/ml).
